# Introgression of Blast Resistance Genes (Putative *Pi-b* and *Pi-kh*) into Elite Rice Cultivar MR219 through Marker-Assisted Selection

**DOI:** 10.3389/fpls.2015.01002

**Published:** 2015-12-17

**Authors:** Fatah A. Tanweer, Mohd Y. Rafii, Kamaruzaman Sijam, Harun A. Rahim, Fahim Ahmed, Sadegh Ashkani, Mohammad A. Latif

**Affiliations:** ^1^Department of Crop Science, Faculty of Agriculture, Universiti Putra MalaysiaSelangor, Malaysia; ^2^Department of Plant Breeding and Genetics, Faculty of Crop Production, Sindh Agriculture University TandojamSindh, Pakistan; ^3^Laboratory of Food Crops, Institute of Tropical Agriculture, Universiti Putra MalaysiaSelangor, Malaysia; ^4^Department of Plant Protections, Faculty of Agriculture, Universiti Putra MalaysiaSelangor, Malaysia; ^5^Agrotechnology and Bioscience Division, Malaysian Nuclear AgencySelangor, Malaysia; ^6^Department of Agronomy and Plant Breeding, Islamic Azad University of Yadegar-e-Imam Khomeini (RAH) BranchTehran, Iran; ^7^Bangladesh Rice Research InstituteGazipur, Bangladesh

**Keywords:** rice, blast resistance, gene pyramiding, MABC, marker-assisted backcrossing, biotic stress

## Abstract

Blast is the most common biotic stress leading to the reduction of rice yield in many rice-growing areas of the world, including Malaysia. Improvement of blast resistance of rice varieties cultivated in blast endemic areas is one of the most important objectives of rice breeding programs. In this study, the marker-assisted backcrossing strategy was applied to improve the blast resistance of the most popular Malaysian rice variety MR219 by introgressing blast resistance genes from the Pongsu Seribu 2 variety. Two blast resistance genes, *Pi-b* and *Pi-kh*, were pyramided into MR219. Foreground selection coupled with stringent phenotypic selection identified 15 plants homozygous for the *Pi-b* and *Pi-kh* genes, and background selection revealed more than 95% genome recovery of MR219 in advanced blast resistant lines. Phenotypic screening against blast disease indicated that advanced homozygous blast resistant lines were strongly resistant against pathotype P7.2 in the blast disease endemic areas. The morphological, yield, grain quality, and yield-contributing characteristics were significantly similar to those of MR219. The newly developed blast resistant improved lines will retain the high adoptability of MR219 by farmers. The present results will also play an important role in sustaining the rice production of Malaysia.

## Introduction

The rice production system makes a vital contribution to the reduction of hunger and poverty. The fast growth of the world population demands an increase of 26% in rice production to fulfill the requirement (Khush, 2013). Rice production has widely increased after the green revolution, but the yield of superior varieties is still not increasing as farmers expect due to the influence of biotic and abiotic factors (Divya et al., 2014). The continuous supply of rice per demand of the consumer can only be achieved by maintaining a stable rice production, which is a challenge for rice breeders (Roychowdhury et al., 2012). Rice production can be managed by introducing new varieties possessing strong resistance against abiotic and biotic factors. Currently, DNA marker technology has immensely contributed to genetic improvement through the selection of desirable traits, such as disease resistance. Molecular markers are a valuable resource in marker-assisted backcross (MABC) breeding to monitor the disease resistance genes. Many rice cultivars resistant to biotic stress have been released and widely adopted by farmers with the application of marker-assisted selection (Xu and Crouch, 2008).

Blast is the one of the main diseases of rice crops causing crop loss in both temperate and tropical rice growing regions (Mackill and Bonman, 1992). The main agent causing this disease is the fungus *Magnaporthe oryzae*. Thus, blast resistance in rice plants has been one of the most important traits being pursued in breeding programs over several decades. Different breeding strategies have been adopted to achieve this serious challenge, such as the use of field resistance to blast disease and the introduction of resistance genes into the rice susceptible cultivar objective (Kushibuchi, 1997). As a result, several blast resistance rice varieties were introduced, but within a short period of time, they have become seriously blast susceptible because of the emergence of new pathotypes of blast fungus. This breakdown of blast resistance clearly indicates that the resistance cannot be widely achieved until true resistance genes are identified (Hittalmani et al., 2000). The continuous identification of resistance genes against blast can lead to genetic control over the new existing pathogens. To date, approximately 347 QTLs linked to blast resistance (Koide et al., 2009; Ballini et al., 2013) and more than 100 blast resistant genes have been identified from diversified rice germplasm (Divya et al., 2014). The identified blast *R* genes were found on all 12 rice chromosomes except 3, and most of them were in a cluster on chromosomes 6, 11, and 12 (Yang et al., 2013). *Pi-b* and *Pi-kh* have been used extensively in rice breeding programs in Japan, China, and Indonesia and are considered to be major blast resistance genes along with *Pi-ta*. *Pi-b* and *Pi-kh* are dominant major blast resistance genes conferring broad spectrum resistance to various isolates of the fungal pathogen *M. oryzae* (Wang et al., 1999; Sharma et al., 2005; Tanweer et al., 2015b). *Pi-kh* has been identified in many tropical Japonica varieties such as Tetep, and *Pi-b* in *Indica* varieties such as Thoku 11 9 (Conaway-Bormans et al., 2003). The dominant gene *Pi-b*, which confers high resistance to most Japanese blast races, has been mapped to the distal end of the long arm of chromosome 2 (Shinoda et al., 1971) and *Pi-kh* to the long arm of rice chromosome 11 (Sharma et al., 2005). The utilization of both of these blast resistance genes in marker assisted-selection breeding programs has been widely observed (Tanweer et al., 2015a).

Marker-assisted backcrossing has enormous potential to introduce the blast resistance genes into diverse rice cultivars (Collard and Mackill, 2008; Collard et al., 2008). Introgression of blast resistant genes into advanced improved rice lines is a cost-effective and environmentally friendly approach to combat yield losses (Wen and Gao, 2012). The main advantage of marker-assisted selection is the accuracy of selection of the true plant within the short breeding cycle to produce blast resistant rice varieties. Currently, the blast resistant breeding program has achieved greater success with the advent of marker-assisted selection (Ragimekula et al., 2013). Recently, blast resistance genes *Piz5* and *Pi54* have been introgressed into the genetic background of the PRR78 rice variety from donor parent C101A51 and Tetep, and blast resistant lines have been developed with the application of MABC breeding (Singh et al., 2012). The selection was based on foreground markers RM287 and RM206 by following repetitive backcrossing. The *Pi1* leaf blast resistance gene has been introgressed into the D521 line derived from the donor line BL122 (Fu et al., 2012). With the application of MABC, 304 elite parental lines of hybrid rice have also been improved with bacterial blight and blast resistance genes (Zhou et al., 2003). Recently, IR64 cultivar submergence tolerant gene *Sub1* has been introgressed into the OM1490 variety (Lang et al., 2011). The QTL *Saltol* derived from a salt tolerant variety also has been introgressed into popular cultivars of Vietnam (Huyen et al., 2012). These examples provide a great opportunity to develop blast resistant rice varieties through MABC breeding. In the present study, the MABC technique was applied to introgress blast resistant genes from the highly resistant rice variety Pongsu Seribu 2 to blast susceptible Malaysian rice cultivar MR219.

## Materials and Methods

### Developing Blast Resistant Lines

The crossing was performed between the parental lines of Pongsu Seribu 2 and MR219, and the F_1_ hybrid was produced (Supplementry Figure S1). After confirming the hybridity of the plants, true hybrid heterozygous plants were backcrossed with recurrent parent MR219, and BC_1_F_1_ generation seeds were produced. Foreground selection for the desired alleles and background selection for the recovery of the recurrent parent were performed. The plants with the desired allele and maximum recovery of recurrent parents were again backcrossed, and subsequently the BC_2_F_1_ generation was produced. The same steps were followed for the BC_2_F_1_ generation, and the best plants were selfed to produce the BC_2_F_2_ generation seed. The complete genome genotyping with SSR markers was performed, distributed over all 12 rice chromosomes. The true plants on the basis of genotype with the desired alleles were backcrossed in each generation. In every generation, the plant showing the heterozygous allele for Pongsu Seribu 2 was selected. At the final stage, 15 homozygous plants carrying target alleles along with a similar genome of MR219 in each chromosome were selected in the BC_2_F_2_ generation, and blast resistant lines were produced.

### Microsatellite Analysis

#### Markers for Foreground Selection

The robust tightly linked marker RM208F 5′-tctgcaagccttgtctgatg-3′, RM208R 5′-taagtcgatcattgtgtggacc-3′ on chromosome 2 linked to the *Pi-b* gene (Wang et al., 1999) and RM206 5′-cccatgcgtttaactattct-3′, RM206R 5′-cgttccatcgatccgtatgg-3′ on chromosome 11 linked to the *Pi-kh* gene (Sharma et al., 2005) were used for selecting the target genes.

#### Markers for Background Selection

A total of 72 polymorphic markers were identified from 300 SSR markers with known chromosomal position covering all 12 rice chromosomes. SSR markers unlinked to the target gene covering the entire chromosome, including carrier chromosomes 2 and 11 that were polymorphic between the recurrent and donor parent, were used for the background selection to recover the recurrent parent genome. At least five polymorphic markers per chromosome were used to generate the data. The assessment of the recovery of the recurrent parent genome was based on the selection of SSR marker data that was carried out by using the software program Graphical Geno Types (GGTs) version 2.0.

#### DNA Extraction

Total genomic DNA was isolated from 21-days-old young fresh leaves of plants of each backcross generation using the CTAB method as mentioned by Doyle (1990) with minor changes.

#### PCR Amplification

For PCR amplification, the protocol described by McCouch et al. (2002) was adapted. The total volume of the PCR reaction was 15 μl, including 70 ng template DNA, 1.0 μl of forward primer, 1.0 μl of reverse primer, 7.4 μl master mix (premixed containing Taq DNA polymerase, dNTPs, and MgCl_2_) and 4.6 μl nuclease free water. PCR amplification was performed using the touch down PCR program using the following protocol: 94°C for 3 min followed by 10 cycles of 94°C for 30 s, 62°C for 1 min (decreasing 1°C per cycle), and 72°C for 30 s, and 30 cycles of 94°C for 30 s, 52°C for 1 min, 72°C for 2 min, and a final extension at 72°C for 10 min by rapid cooling to 4°C prior to analysis.

#### Gel Electrophoresis

The gel was prepared by mixing 3.0% metaphor^TM^ agarose (Lonza) gel in 1× TBE buffer (0.05 M Tris, 0.05 M boric acid, 1 mM EDTA, pH 8.0). In total, 1 μl Midori green was also added for staining. The gel was run at 80 V for 80 min, and finally the amplified product was visualized in the Molecular imager^®^ (GelDoc^TM^ XR, Bio-Rad Laboratories, Inc., USA).

#### Phenotypic Screening of Plants against *Magnaporthe oryza* Pathoype P7.2

The most virulent pathotype P7.2 of the *M. oryzae* isolate was provided by MARDI (Malaysian Agriculture and Research Development Institute). The plants of the donor parent, recurrent parent and BC_2_F_2_ generations were phenotypically screened in field conditions. The young plants of 21 days were inoculated by spraying spore suspension at a concentration of 1.5 × 10^5^ conidia/ml, and 90% humidity was maintained by covering the plants with plastic bags to develop the disease. The inoculated plants were observed after 9 days of inoculation for blast disease lesions. The plants and blast lesion degrees (BLDs) were evaluated on the basis of 0-9 of the IRRI-SES scale (IRRI, 1996). The percentage of disease leaf area (%DLA) and blast lesion type (BLT) were scored as described by Correa-Victoria and Zeigler (1993). The percentage of DLA was calculated from 0 to 100%. For the BLT score, either 0 (highly resistant: no any symptoms), 1-2 (no sporulation, lesion 1–2 mm), 3 (little sporulation, round lesion), or 4 (heavy sporulation, spindle shaped lesion) were scored. For single-gene model analysis, if the plant showed lesion type 0-3, the plant was considered a resistant plant, and plants showing lesion type 4 or above were considered to be susceptible for the selected pathotype P7.2 in the selected BC_2_F_2_ population. For the two-gene model, blast resistance was classified as resistant (R) (1-2), moderately resistant (MR) (3), moderately susceptible (MS) (4-6) and susceptible (S) (7-9). The protocol of Singh et al. (2013) was followed with minor modification for plant disease reaction. The phenotypic segregation of plants for the two-gene model was calculated as 9:3:3:1 (R: MR: MS: S). A test for an effect of duplicate dominant gene action (epistasis) was analyzed by observing the resistant versus susceptible plants 15(R):1(S) ratio in the BC_2_F_2_ population. The plants showing a disease lesion score of 0-6 were resistant plants and 7-9 were susceptible plants.

#### Agronomic Performance of the Selected Best Lines of the BC_2_F_2_ Generation

The lines having a maximum recovery of the recurrent parent along with target genes and phenotypic similarity with the recurrent parent were used to observe the agro-morphological traits. Different parameters related to yield and yield contributing factors were recorded, such as days to 50% flowering, days to maturity, plant, panicles per plants, effective tillers per plants, panicle length, seed setting rate, full filled grain per plants seed setting rate, 1000-grain weight, yield per plant, grain length, grain width, flag leaf length and flag leaf width (Supplementry Table S1). These traits were recorded from all of the best selected lines of BC_2_F_2_ along with the recurrent parent.

### Statistical Analysis

The BC_2_F_2_ population segregation data were analyzed using a chi-square test (χ^2^). An analysis for goodness of fit to the expected ratio of 3:1, 9:3:3:1, and 15:1 was calculated using the chi square formula χ^2^ = (O-E)^2^/E, where O represents observed value and E is the expected value. Analysis of a single marker was performed using SAS 9.3 software as mentioned by Divya et al. (2014). This analysis of single markers was fitted to the linear regression model: Y- bo+blx+e. The results were obtained as the estimate of *R*^2^ value and the F statistic for each marker. The *R*^2^ significance indicates the linkage of markers with the trait. The SSR genotyping data were analyzed using GGT software 2.0. The mean difference for the selected best lines of BC_2_F_2_ and the recurrent parent MR219 was analyzed using an independent *t*-test in the SAS 9.3 software.

## Results

### Marker-assisted Foreground Selection

**FIGURE 1:**
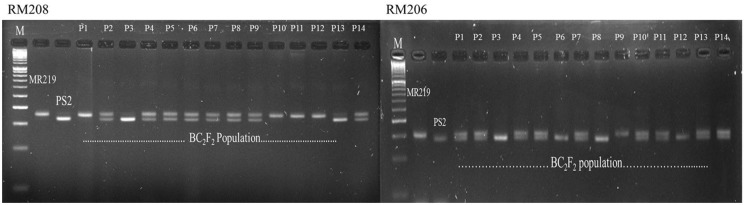
**Genotyping of blast resistant improved homozygous lines using tightly linked marker RM208 and RM206**.

Crosses were made between the parental line of MR219 and Pongsu Seribu 2, and F_1_ seeds were produced. The best F_1_ plants were screened with foreground markers to identify the true F_1_ plants carrying the gene of interest in a heterozygous form. The six gene positive plants were backcrossed with the recurrent parent to generate the next generation BC_1_F_1_ seed. The BC_1_F_1_ plants were screened for the selection of a heterozygous allele at the putative *Pi-b* and *Pi-kh* locus with RM208 and RM206 markers along with phenotypic maximum similarity with the recurrent parent. The allele size in base pairs (bp) of both the parents MR219 and Pongsu Seribu 2 amplified by both markers has been given in Supplementry Table S2. The best plants of BC_1_F_1_ having an appearance similar to MR219 and carrying the target gene were again crossed with the recurrent parent and 320 plants of the BC_2_F_1_ generation were selected. Similarly, BC_1_F_1_ plants were screened to identify the plants in heterozygous form with maximum RPG recovery. Selfing was performed in the BC_2_F_1_ plants, and the BC_2_F_2_ generated seeds were grown and plants with similarity to MR219 along with homozygous resistant alleles using RM208 and RM206 were selected (**Figure 1**). The genotypic segregation of the BC_2_F_2_ population using linked markers RM208 and RM206 is shown in **Table 1**. Both of the markers represent a good fit to the expected marker segregation ratio (1:2:1) according to the expected Mendelian ratio. From this selection, 15 best improved blast resistant lines were evaluated and selected.

**Table 1:** Analysis of markers in BC_2_F_2_ segregating population.

Markers	Marker segregation analysis			χ^2^(1:2:1)	Probability
	AA = R	AB = SG	BB = S		
RM208	53	106	41	2.16	0.339
RM206	55	107	38	3.87	0.144

*According to model on single dominant gene, (AA): Resistant; (BB): Susceptible; and (AB): Segregant. df = 2; χ^*2*^(0.05,2) = 5.99*.

### Screening against Blast Disease in MR219 and Pongsu Seribu 2

**FIGURE 2:**
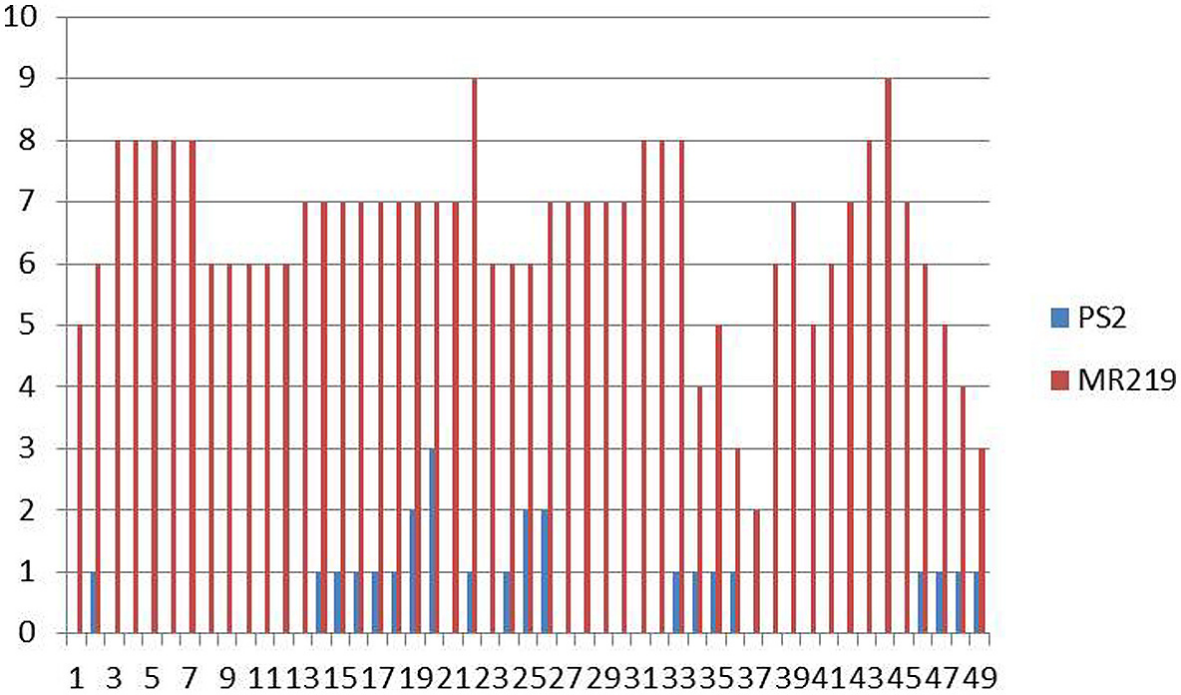
**Distribution of blast lesion degree in the parental line of MR219 and Pongsu Seribu 2**.

Pongsu Seribu 2, the donor parent having *Pi* genes, expressed a strong spectrum of resistance against pathotype P7.2 with a score of 0-1 while the recurrent parent showed susceptibility with a score of 5-9 (**Figure 2**). The blast disease reaction is shown in Supplementry Figure S2.

### Screening against Blast Disease in Improved Blast Resistant Lines of the BC_2_F_2_ Population

**FIGURE 3:**
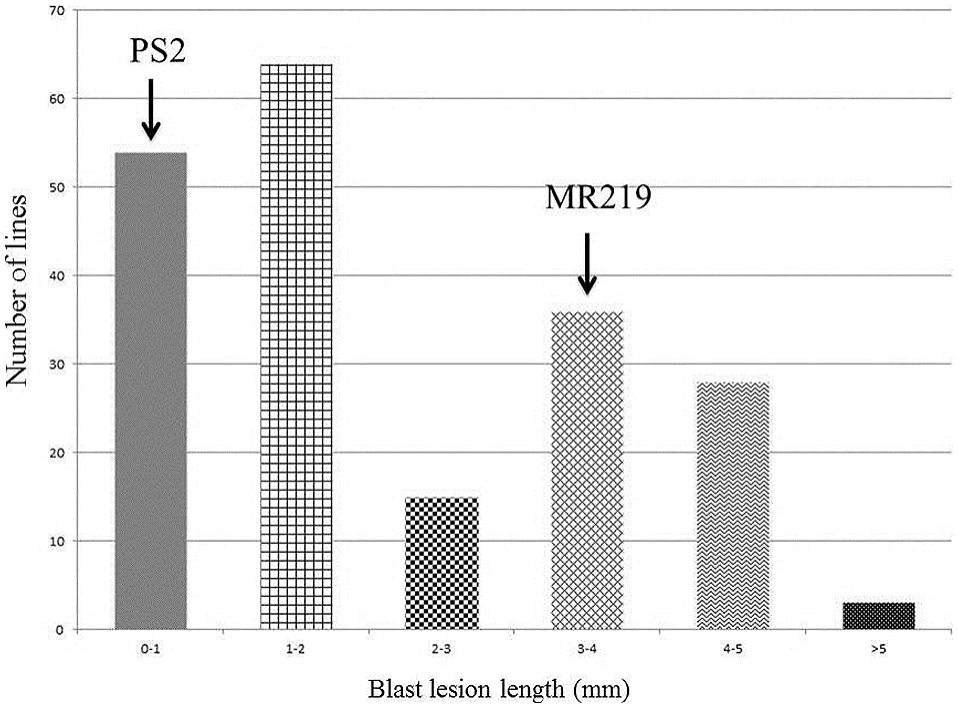
**Calculation of the distribution of blast lesion length after inoculation with *Magnaporthe oryzae* pathotype P7.2 in parental lines along with improved introgressed blast resistant gene lines of the BC_2_F_2_ population (*n* = 200)**.

The advance 15 improved MR219 lines carrying blast resistance genes produced from both of these parents, 5-3-7-1, 5-3-7-4, 5-3-7-13, 5-3-7-19, 5-3-7-24, 5-3-7-29, 5-3-7-31, 5-3-7-36, 5-3-7-40, 5-3-7-69, 5-3-17-2, 5-3-17-4, 5-3-17-11, 5-3-17-19, 5-3-17-21, showed a great magnitude of resistance with a score of 0-1. The distribution of the BLD treated with *M. oryzae* pathotype P7.2 in the parental lines and advanced improved lines with introgressed blast resistant genes of the BC_2_F_2_ populations is summarized in **Figure 3**.

### Assessment of the Phenotypic Segregation of Blast Resistant versus Susceptible Plants

A chi-square test was performed to evaluate the blast disease in the BC_2_F_2_ families. Different models, i.e., the single-gene model, two independent gene model and interaction of two different loci (Epistasis) were tested to assess which model population fit to the expected ratio. The number of expected resistant and susceptible plants for the phenotypic segregation ratio was not significantly different from the number of the observed resistant and susceptible plants and followed the expected Mendelian (3:1) ratio (**Table 2**).

**Table 2:** Phenotypic segregation ratio of observed and expected number of resistant and susceptible plants in the BC_2_F_2_ population inoculated with highly virulent pathotype P7.2 of *Magnaporthe oryzae.*

Disease reaction	No. of observed plants	Expected No.	χ^2^(3:1)	*P*-value
Resistant	161	150	0.389	
Susceptible	39	50	1.36	
Total	200	200	1.749	0.0724

*df = 1, χ^*2*^(0.05,1) = 3.84*.

The BC_2_F_2_ population did not follow the two-gene model. Phenotypic disease segregation of the BC_2_F_2_ population did not show a good fit to the expected 9:3:3:1 ratio (**Table 3**). The present results do not support the idea of the two-gene model, thus indicating that resistance to blast in the BC_2_F_2_ generation was not regulated by two different genes. Similarly, the chi-square value for an epistatic effect of the resistant versus susceptible plants does not segregate into 15:1 (**Table 3**) for the BC_2_F_2_ population, therefore the epistatic/two locus interaction was absent.

**Table 3:** Chi-square test for independent gene model (9:3:3:1) and epistatic effect (15:1) for blast resistance in BC_2_F_2_ population inoculated with pathotype P7.2 of *Magnaporthe oryzae.*

Gene model	Observed ratio				Expected ratio	χ^2^ value	*P*-value
	R	MR	MS	S			
Two gene	99	62	20	19	9:3:3:1	29.173	<0.0001
Epistatic effect	165	–	–	35		43.200	<0.0001

*χ^*2*^(0.05,3) = 7.81, df = 3, χ^*2*^(0.05,1) = 3.84, df = 1; R, resistant; MR, moderately resistant; MS, moderately susceptible; S, susceptible*.

### Marker-trait Association

Marker-trait association was analyzed by using SAS 9.3 software to identify the association among the resistance component, i.e., BLD, BLT and %DLA, with tightly linked polymorphic markers of corresponding blast resistance genes. Data of genotypic segregation of linked SSR markers obtained from the BC_2_F_2_ population were combined with phenotypic segregation data of the BC_2_F_2_ population for blast resistance traits. The data were interrogated to determine the significance level and linear model regression analysis for association between the marker genotypes and resistance component traits. The markers RM208 and RM206 showed significant association with BLD, percentage diseased leaf area (%DLA) and BLT with a simple linear regression (*R*^2^) value of more than 10 (**Table 4**).

**Table 4:** Association between the marker and trait in the BC_2_F_2_ population analysed by regression analysis.

Traits	Markers	*R*^2^ (%)
BLD	RM208	25.93^∗∗^
	RM206	19.6^∗∗^
BLT	RM208	15.99^∗∗^
	RM206	13.4^∗∗^
%DLA	RM208	24.3^∗∗^
	RM206	18.6^∗∗^

*^∗∗^Significance at 0.01 level, BLD, blast lesion degree; %DLA, percentage diseased leaf area; BLT, blast lesion degree*.

### Variation and Correlation among Traits

The trait variations (means of the parents and BC_2_F_2_ population) for pathotype P7.2 are shown in **Table 5**. The average leaf blast disease severity score for recurrent parent MR219 was 6.63 for BLD, 3.43 for BLT and 61.43% for DLA. For the donor parent Pongsu Seribu 2 cultivar, the leaf blast disease severity score was 1.92 for BLD, 2.34 for BLT, and 12.4% for DLA. The parental cultivar showed a significantly different (*P* < 0.01) leaf blast resistance. In the BC_2_F_2_ population, the score for BLD, BLT, and DLA was 4.48, 2.5, and 47.18% with standard deviations of 2.24, 1.17, and 22.54, respectively. The selected best 15 lines from the BC_2_F_2_ population showed strong resistance against leaf blast for specific pathotype P7.2. The mean of disease severity of the selected improved lines for BLD, BLT, and DLA was 0.97, 0.88, and 3.96%. The disease severity showed a strong correlation among resistance components BLD, BLT, and %DLA in the BC_2_F_2_ families (**Table 6**).

**Table 5:** Trait variation for selected pathotype P7.2 of *Magnaporthe oryzae* inoculated in BC_2_F_2_ population.

Traits	Means of parents		BC_2_F_2_ population (*n* = 200)		Selected best plants (*n* = 15)	
	MR219 (*n* = 100)	P.Seribu 2 (*n* = 100)	Mean	*SD*	Mean
BLD	6.63	1.92	4.48	2.24	0.97
BLT	3.42	2.34	2.5	1.17	0.88
%DLA	61.43	12.4	47.18	22.54	3.96

**Table 6:** Correlation coefficient between BLD, BLT, and %DLA for pathotype P7.2 in BC_2_F_2_ population.

Traits	BLD	BLT	%DLA
BLD	0.0		
BLT	89.35^∗∗^	0.0	
%DLA	98.53^∗∗^	88.28^∗∗^	0.0

*^∗∗^Correlation is significant at the 0.01 level (two tailed)*.

### Recovery of the Recurrent Parent Genome in Selected Improved Homozygous Lines

**FIGURE 4:**
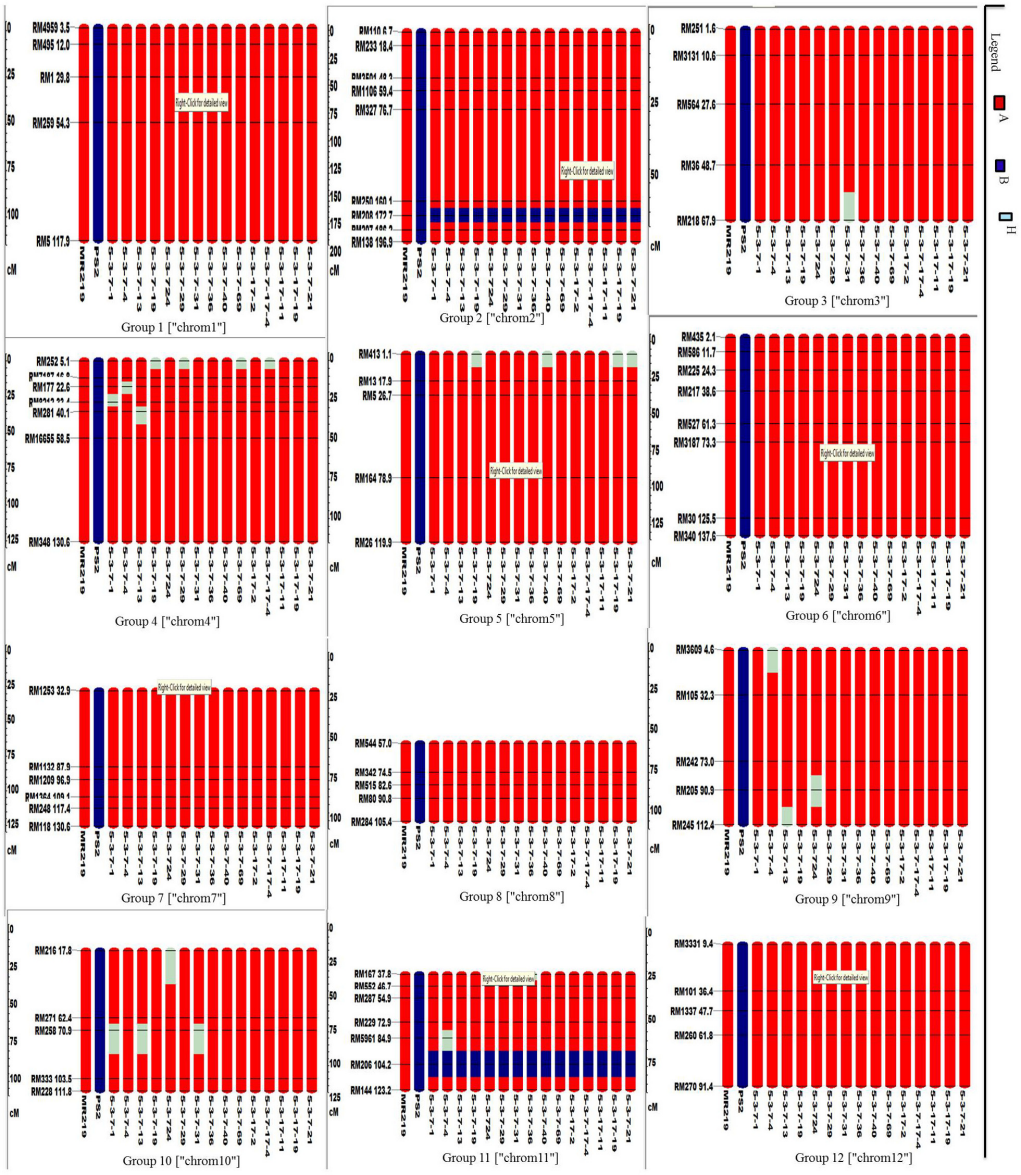
**Graphical genotyping of selected 15 improved lines with introgressed *Pi* genes along with MR219 background developed in this study**. The red color indicates homozygous regions for MR219, the blue color indicates homozygous regions for Pongsu Seribu 2 and the light green color indicates heterozygous regions.

**FIGURE 5:**
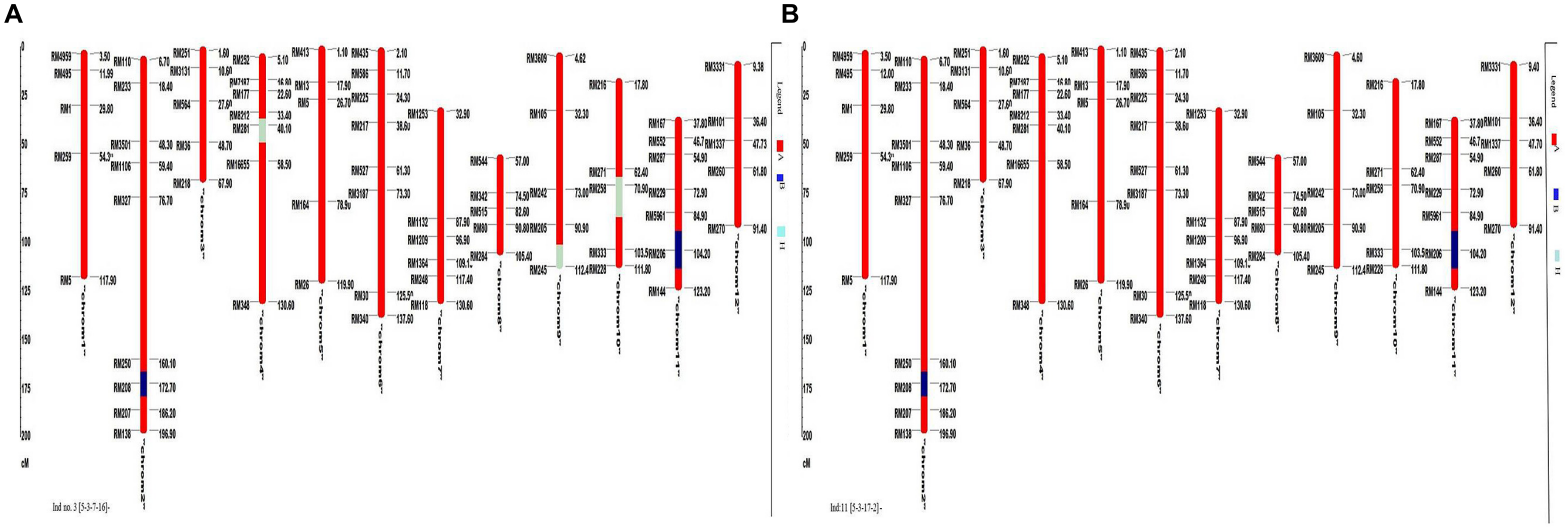
**Graphical genotyping of the improved lines with the lowest **(A)** and highest **(B)** recovery among the best 15 improved lines**. The red color indicates homozygous regions for MR219, the blue color indicates homozygous regions for Pongsu Seribu 2 and the light green color indicates heterozygous regions.

A total of 72 markers were used for background and the selection of improved blast resistant lines, and a genetic map was constructed covering 1266 cM with an average marker distance of 15.91 regions of the whole genome of *Oryza sativa*. A graphical representation of the carried chromosome 2 (putative *Pi-b*) and chromosome 6 (putative *Pi-kh*) of the selected improved blast resistance lines is shown in **Figure 4**. The minimum recovery of the recurrent parent genome in an improved lined was 94% and the maximum recovery in an improved line was 97.5% (**Figure 5**). Most of the residual segments from donor genome content were distributed on chromosomes 4, 9, and 10; however, other chromosomes were completely recovered. The percentage of chromosome segments derived from Pongsu Seribu 2 was 2.5% and remained constant in all of the advanced improved lines. The average proportions of the recurrent parent genome in all 15 improved lines were 96.17%, showing the maximum similarity observed at the phenotypic level with the recurrent parent (**Table 7**).

**Table 7:** Introgressed and background recovery analysis in selected improved lines.

Improved individuals	A (%)	B (%)	H (%)	Total (cM)	H-segment
5-3-7-1	95.1	2.5	2.3	1266	2
5-3-7-4	94.5	2.5	3	1266	3
5-3-7-13	94	2.5	3.5	1266	3
5-3-7-19	96.3	2.5	1.1	1266	1
5-3-7-24	94.1	2.5	3.3	1266	2
5-3-7-29	97	2.5	0.5	1266	1
5-3-7-31	95.1	2.5	2.4	1266	2
5-3-7-36	97.1	2.5	0.4	1266	1
5-3-7-40	96.8	2.5	0.7	1266	1
5-3-7-69	97	2.5	0.5	1266	1
5-3-17-2	97.5	2.5	0	1266	0
5-3-17-4	97	2.5	0.5	1266	1
5-3-17-11	97.5	2.5	0	1266	0
5-3-17-19	96.8	2.5	0.7	1266	1
5-3-17-21	96.8	2.5	0.7	1266	1
Average	96.17	2.5	1.30	1266	1.33

*A = Recurren; B = Donor; H = Heterzygous; cM, Centimorgan*.

### Comparison of Agro-morphological Performance of Improved Lines versus Recurrent Parent MR219

Agro-morphological traits of the advanced improved lines carrying additional blast resistant genes were measured and compared with the recurrent parent lines of MR219 (**Table 8**). There was minor variation in the days to flowering and total grain per panicles. However, other improved lines showed mostly similar performance and there was no significant differences found for other traits, such as plant height, days to maturity, total tiller/hill, effective tiller/hill, panicle length, no. of filled grains/panicle, seed setting rate, 1000 grain weight, yield/plant, grain length, grain width, flag leaf length, and flag leaf width.

**Table 8:** Performance of major agronomic traits of BC_2_F_2_ improved lines carrying blast resistant (putative *Pi-b* and *Pi-kh*) gene.

Traits	MR219(recurrent parent)	BC_2_F_2_ improved lines
Days to 50% flowering (day)	88.53 ± 0.36	87.66 ± 0.33
Plant height (cm)	95.3 ± 0.31	95.8 ± 0.24
Days to maturity (day)	117.8 ± 0.26	117.5 ± 0.25
Total tiller/Hill (no)	17.00 ± 0.30	17.26 ± 0.28
Effective tiller/Hill (no)	16.40 ± 0.28	16.5 ± 0.25
Panicle length (cm)	25.20 ± 0.20	25.60 ± 0.23
Total grain/panicle (no)	166.9 ± 1.46	167.5 ± 1.36
Seed setting rate (%)	90.33 ± 0.23	90.26 ± 0.34
1000 grain weight (gm)	25.88 ± 0.24	26.23 ± 0.13
Yield/plant (gm)	42.06 ± 0.28	42.26 ± 0.26
Grain length (mm)	9.71 ± 0.02	9.74 ± 0.03
Grain width (mm)	1.98 ± 0.029	2.00 ± 0.03
Grain length/width	4.92 ± 0.07	4.94 ± 0.09
Flag leaf length (cm)	33.86 ± 0.29	33.53 ± 0.27
Flag leaf width (cm)	1.5 ± 0.003	1.52 ± 0.27
No. of filled grain/panicle	154.1 ± 1.35	154.4 ± 1.08

*Significance at 5% level with independent *t*-test*.

## Discussion

Rice production is always constrained by several biotic stresses, among which blast diseases impose both several yield and quality losses. These serious and most challenging issues could be overcome by utilizing resistance genes (Tabien et al., 2002). Pyramiding major resistance genes into elite rice cultivars with the application of conventional breeding were always hindered by environmental factors and the number of generations needed to achieve the goal. However, marker-assisted selection saves time and offers a very simple, efficient, and accurate method to improve the blast resistance of elite genotypes (Singh et al., 2012). The linkage drag can be minimized within a few generations, and the recurrent parent genotype can be stored easily with additional genes of interest (Joseph et al., 2004; Shanti et al., 2010). However, in the backcross breeding program, the choice of the recurrent parent plays a vital role (Ye and Smith, 2010). Blast resistance provided by identified genes is always race specific against the pathotype. Ultimately, the resistance of most of the varieties is lost due to variability of pathogenicity of the pathotype. The blast pathotypes of *M. oryzae* are able to change their virulence according to the environment. Therefore, the identification of more closely linked markers with blast resistance genes can help to introgress identified genes into improved cultivars through marker-assisted selection. The closely linked marker helps to monitor blast resistance genes for several generations (Jena and Mackill, 2008). Breeders have reduced the yield loss due to blast disease by introgressing the beneficial alleles from the wild rice genotype into elite high yielding rice cultivars (Brar and Khush, 1997). DNA marker technology has greatly facilitated the tagging of novel resistance genes from wild rice species and provides a straight forward way to identify and transfer the major genes from unadapted germplasm to adapted germplasm (Gu et al., 2013).

The present research was conducted to improve the blast resistance of the elite Malaysia rice variety MR219 through a MABC breeding approach along with phenotypic selection for agro-morphological traits. From previous studies, the IR64 rice variety was improved for blast resistance coupled with phenotypic selection for agro-morphological traits similar to our study (Sreewongchai et al., 2010). By using the MABC strategy, improved versions of the elite Basmati variety, Pusa Basmati 1, Pusa RH10 and KMR-3R were also released for bacterial blight resistance (Gopalakrishnan et al., 2008; Basavaraj et al., 2010; Hari et al., 2011). Narayanan et al. (2002) introgressed *Piz-5 blast* resistance gene into rice cultivar IR50 and improved the blast resistance. Singh et al. (2012) introgressed the blast resistance gene *Pi-54* (previously known as *Pi-kh*) into Pusa Basmati 1 from donor parent Tetep. This is first report in Malaysia documenting the stacking of two major genes (*Pi-kh* and *Pi-b)* in elite rice cultivar MR219 through MABC breeding coupled with phenotypic selection for agro-morphological traits.

For PCR-based DNA markers used in the present study, RM208 tightly linked with the *Pi-b* gene (Wang et al., 1999; Roychowdhury et al., 2012) and RM206 tightly linked with the *Pi-kh* gene (Sharma et al., 2005; Singh et al., 2012; Hari et al., 2013). The RM208 marker presents on chromosome 2 and RM206 on chromosome 6 below the centromere. Both of these markers are highly polymorphic and can be detected very easily and therefore have great potential to serve as an important tool to introgress *Pi-b* and *Pi-kh* blast resistant genes into blast susceptible rice varieties. The importance and benefit of using tightly linked markers for gene pyramiding have been discussed earlier by Hittalmani et al. (2000) and Hayashi et al. (2006) for blast disease screening. However, the success of marker-assisted selection heavily depends upon the strong linkage between the marker and target gene. Thus, from the blast disease screening results, 15 best selected lines, 5-3-7-1, 5-3-7-4, 5-3-7-13, 5-3-7-19, 5-3-7-24, 5-3-7-29, 5-3-7-31, 5-3-7-36, 5-3-7-40, 5-3-7-69, 5-3-17-2, 5-3-17-4, 5-3-17-11, 5-3-17-19, 5-3-17-21, showed strong resistance against virulent pathotype P7.2 similar to the donor parent. Among the introgressed lines, BC_2_F_2_ with genes *Pi-b* and *Pi-kh* showed high resistance at both locations. The results of the phenotypic screening against blast disease reaction of the improved lines carrying the putative *Pi-b* and *Pi-kh* genes with a background of the recurrent parent MR219 conferred complete resistance to the highly virulent pathotype P7.2, indicating the strong bond between these markers with the trait.

Seventy-two polymorphic SSR markers between parental lines with at least five markers per chromosome were used for genetic background selection. Most of the recurrent parent segments were fully recovered in improved lines, but in some improved lines, some chromosomes were not recovered. Some heterozygous segments were found in some improved lines. The results are consistent with the finding of Tian et al. (2006) and Hirabayashi et al. (2010), who described that some regions of the BC_2_F_2_ generation may not be fully recovered if the marker-assisted selection is not performed until the BC_3_F_2_ generations. Some other biological factors may also be involved, such as gametophyte, heading date and hybrid sterility (Doi et al., 1997). Recurrent parent phenotype can be recovered in one or two backcrosses if more than one resistance gene is transferred from *indica* to *japonica* cultivars (Singh et al., 2001; Rajpurohit et al., 2011). Similarly, Shu (2009) transferred multiple resistance genes, such as *Xa4, xa5 and Xa21*, from the *indicia* cultivar to the *japonica* cultivar for BB resistance and mentioned that at least three backcrosses are required to recover the recurrent parent phenotype. In this study, a similar approach was adopted for foreground and background selection for higher recovery of the background genotype and introgression of target genes in the *indica/indica* cultivar in two backcrossed and one self-generation. This strategy is very effective in minimizing the cost and time required to recover the desirable recombinants to a considerable extent with target resistance genes in the *indica/indica* crosses.

The donor parent Pongsu Seribu 2 and the recurrent parent MR219 showed significantly different agro-morphological traits. However, in the blast resistant, improved lines of MR219, no apparent yield penalty was related with the presence of the blast resistance genes, and putative *Pi-b* and *Pi-kh* were observed. Therefore, the cultivation of our improved blast resistant lines would be of great advantage to reduce the yield losses in blast disease endemic areas. The introgression of blast resistance genes along with nearly complete recovery of the genome of the recurrent parents in improved advanced MR219 lines and yield is the greatest achievement of the current research. Yield and grain quality traits are multigenically encoded by loci that are distributed throughout the rice genome (Sundaram et al., 2008). In this study, a higher recovery of desirable improved plants of MR219 was obtained because of phenotypic-based selection for agro-morphological traits from the BC_1_F_1_ generation onward and screening of a quantifiable number of BC plants. The current strategies of phenotypic-based along with marker-based selection are consistent with results of Joseph et al. (2004) and Gopalakrishnan et al. (2008), who adopted the phenotypic-based selection for grain type and molecular-based for the target trait (i.e., bacterial blight and blast resistance). Improved blast resistant MR219 lines showed a similar agro-morphological performance in the field as a par recurrent parent MR219 with a minor acceptable difference. The mean value of blast resistance lines (carrying the *Pi-b* and *Pi-kh* genes) for all morphological characters were mostly similar with the recipient parent MR219, indicating that the performance of introgression lines is similar with MR219 for such traits. The present results strongly support that our phenotypic selection practice was efficient. These results are also consistent with the finding of Yoshimura et al. (1995) and Steele et al. (2006) who found that the offspring of resistant parental lines shows a similar or better level of resistance and has preferable quality and yield characteristics for further selection. Considering the agro-morphological traits, there was a significant difference for days of 50% flowering; some lines took the same time as MR219 and some lines were delayed in flowering. In MR219, flowering was significantly earlier under the proper irrigation (Rahim et al., 2012). The late flowering in some introgression lines was due to donor parent Pongsu Seribu 2, which takes more time compared to MR219. For the grain yield per plant, there was not any significant difference among the parental line and integration line. The present finding is similar to Sabu et al. (2006), who also did not find any significant difference in grain yield of parent lines and advanced backcrossed lines. The entire advanced breeding line (ABL) grain characteristics were similar to the recurrent parent (MR219). The number of panicles depends on the effective tillers number; if there are more effective tillers, there will be more panicles (Hossain et al., 2008). Biswas et al. (1998) also studied the genotypic difference of grain yield and reported that higher grain yield depended on the number of effective tillers per hill and number of grains per panicle. Shi et al. (2000) also reported that the exterior quality of the rice grain depends on the grain length and width. The proportion of the grain length to width in all backcross introgression lines and MR219 was a slender grain shape. According to Rafii et al. (2014), high grain length with low grain width could lead to a long shaped grain. Grain shape is controlled by triploid endosperm, cytoplasmic and maternal genes.

Until now, most of the breeders have introgressed a single major gene into blast susceptible varieties. The high level of instability in the pathogen genome could lead to break-down of the resistance based on the single gene (Hittalmani et al., 2000). The best way to sustain the resistance for long term is the incorporation of partial resistance or combining putative QTL or incorporating multiple genes that decrease the selection pressure on the pathogen, thus resistance remains for a long time (Bonman, 1992; Tabien et al., 2002; Lopez-Gerena, 2006). However, pyramiding major resistance genes into a single cultivar will be effective for a particular set of virulent pathotypes (Hittalmani et al., 2000). Ultimately, rice cultivar with durable resistance by accumulating major genes and QTL for partial resistance against *M. oryzae* is an ideal strategy to control blast disease.

## Conclusion

The present study suggests that DNA markers for blast resistance (putative *Pi-b* and *Pi-kh*) genes are reliable for marker-assisted selection of blast resistance in rice breeding. The recovery of the recurrent parent along with the intogression of blast resistance enes with MABC breeding was much faster than that with conventional breeding. Fifteen improved blast resistance lines were produced from a backcross between the parental line MR219 and Pongsu Seribu 2. These improved blast resistant lines could be utilized as a source of genetic material for blast resistance with a high yielding background of MR219. The introgressed resistant genes *Pi-b* and *Pi-kh* are dominant blast resistance genes; therefore, the resistance in improved blast resistant lines will remain for long periods, thus enhancing the food security in Malaysia. These improved blast resistant lines have a practical breeding value without yield penalty by providing blast resistance against the highly virulent pathotype P7.2 that exists in Malaysia. Identifying the most resistant lines will lead to durable resistant rice varieties and serve as a source of genetic resistance in the rice germplasm, which will have a great impact on the rice yield sustainability and stability. To our knowledge, this is the first report on the successful introgression of blast resistant genes (*Pi-b* and *Pi-kh*) into the elite high yielding rice cultivar MR219 in Malaysia.

## Conflict of Interest Statement

The authors declare that the research was conducted in the absence of any commercial or financial relationships that could be construed as a potential conflict of interest.
