# Supplementary material for: Introgression of Blast Resistance Genes (Putative Pi-b and Pi-kh) into Elite Rice Cultivar MR219 through Marker-Assisted Selection
Source: Front Plant Sci. 2015 Dec 17;6:1002. doi: 10.3389/fpls.2015.01002 (PMC4682138; doi:10.3389/fpls.2015.01002)
Supplement: Supplementary file 1 [file Data_Sheet_1.DOCX]

**Supplementary Tables**

**Table S.1.** **Description of the Agro-morphological traits measured in parental lines along with BC_2_F_2_ generation plants**

| **Trait** | **Unit of measurement** | **Method of evaluation** |
| --- | --- | --- |
| Days to 50% flowering | Day | Days from sowing in field to 50 panicles heading from each plant. |
| Plant height | Centimeter | Plants height was measured from ground to the tip of the tallest flat leaf. |
| Days to maturity | Day | Days from sowing in field to 80% grains reaching golden yellow. |
| Total tiller per plant | No. per plant | All tillers were counted from each plant. |
| Effective tillers per plant | No. per plant | Average tiller number those had panicle with grain. |
| Panicle length | Centimeter | From the base (first node) to the tip of last spikelet of panicle (excluding awn). |
| Total grain per panicle | No. | Counting the total spikelet (filled and unfilled grains) per panicle. |
| Filled grains per panicle | NO. | Subtracted unfilled grain from total grains of a panicle. |
| Seed setting rate | Percent (%) | (number of grain per panicle)/ (Number of spikelet per panicle) multiply by 100. |
| 1000-grain weight | Gram | One thousands full filled seeds counted and weighted. |
| Yield per plant | Gram per plant | Harvested all grains from each plant and weighted. |
| Grain length | Millimeter | Longitudinal dimension measured from the base of the lower most sterile lemma to the tip (apiculus) of the lemma or pales. |
| Grain width | Millimeter | Dorsoventral diameter measured from well-developed grains as the distance across the lemma and the palea at the widest point. |
| Flag leaf length | Centimeter | The top most leaf below the panicle is the flag leaf length was measured from base to the tip of flag leaf. |
| Flag leaf width | Centimeter | Width was measured from middle widest point of flag leaf. |

**Table S.2.** **Allele size of the foreground markers linked to blast resistant genes (Putative *Pi-b* and *Pi-kh*) in susceptible (MR219) and resistant (Pongsu Seribu 2) parents**

| Markers | Chromosome | Linked gene | Alleles in base pairs (bp) | |
| --- | --- | --- | --- | --- |
|  |  |  | Susceptible parent | Resistant parent |
| RM208 | 2 | *Pi-b* | 182 199 | |
| RM206 | 6 | *Pi-kh* | 151 142 | |

**Supplementary Figures Caption**

**Figure S.1.** Procedure of marker-assisted backcrossing for producing an improved blast resistant rice line.

**Figure S.2.** Blast disease reaction, inoculated with the virulent pathotype P7.2 of *Magnaporthe oryzae*. a= challenging with pathotype P7.2, b= blast lesion degree in the BC_2_F_2_ population after inoculation.
