# Supplementary figures and images for: Introgression of Blast Resistance Genes (Putative Pi-b and Pi-kh) into Elite Rice Cultivar MR219 through Marker-Assisted Selection
Source: Front Plant Sci. 2015 Dec 17;6:1002. doi: 10.3389/fpls.2015.01002 (PMC4682138; doi:10.3389/fpls.2015.01002)

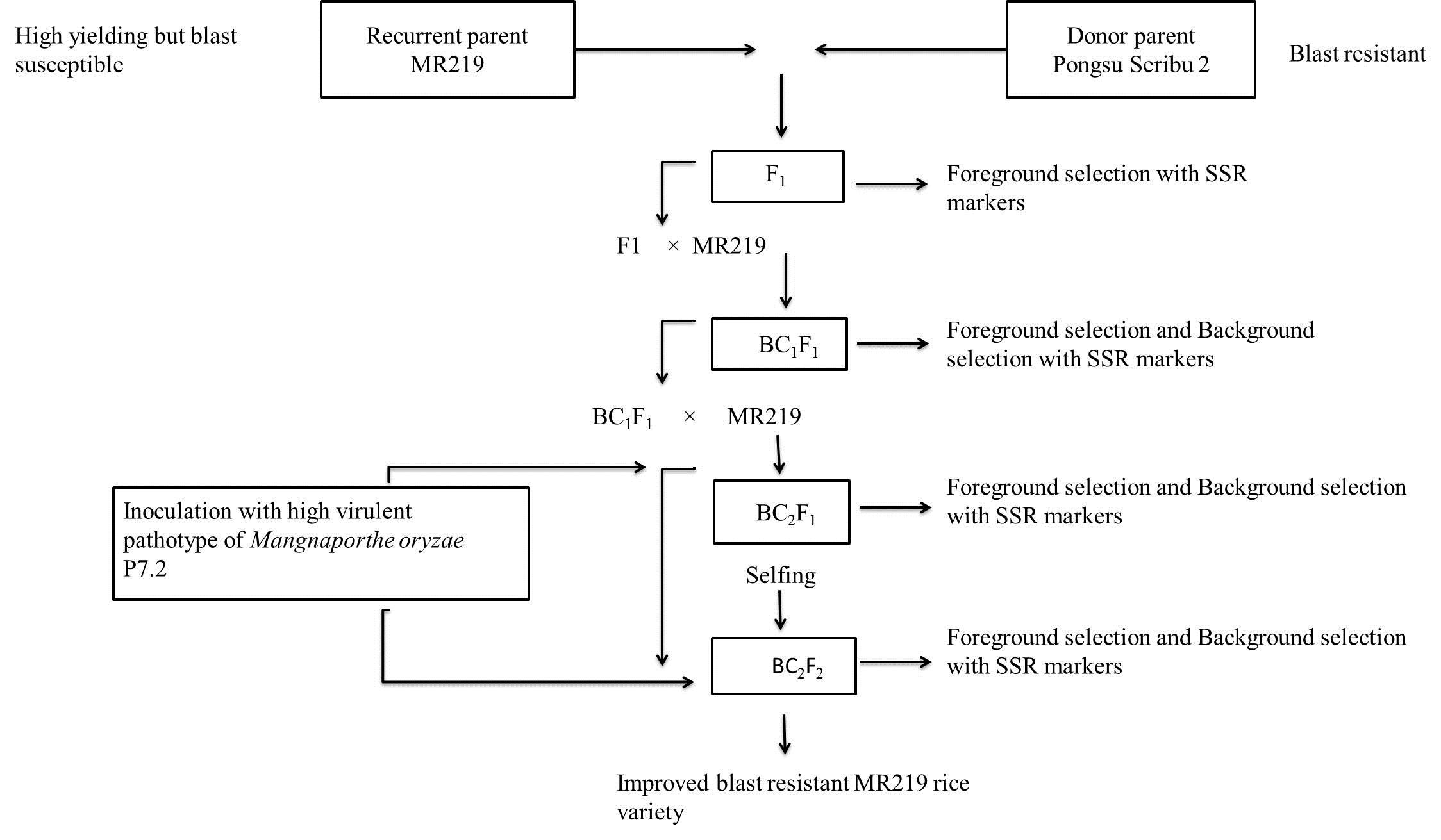

Supplement: Supplementary file 2 [file Image_1.JPEG]

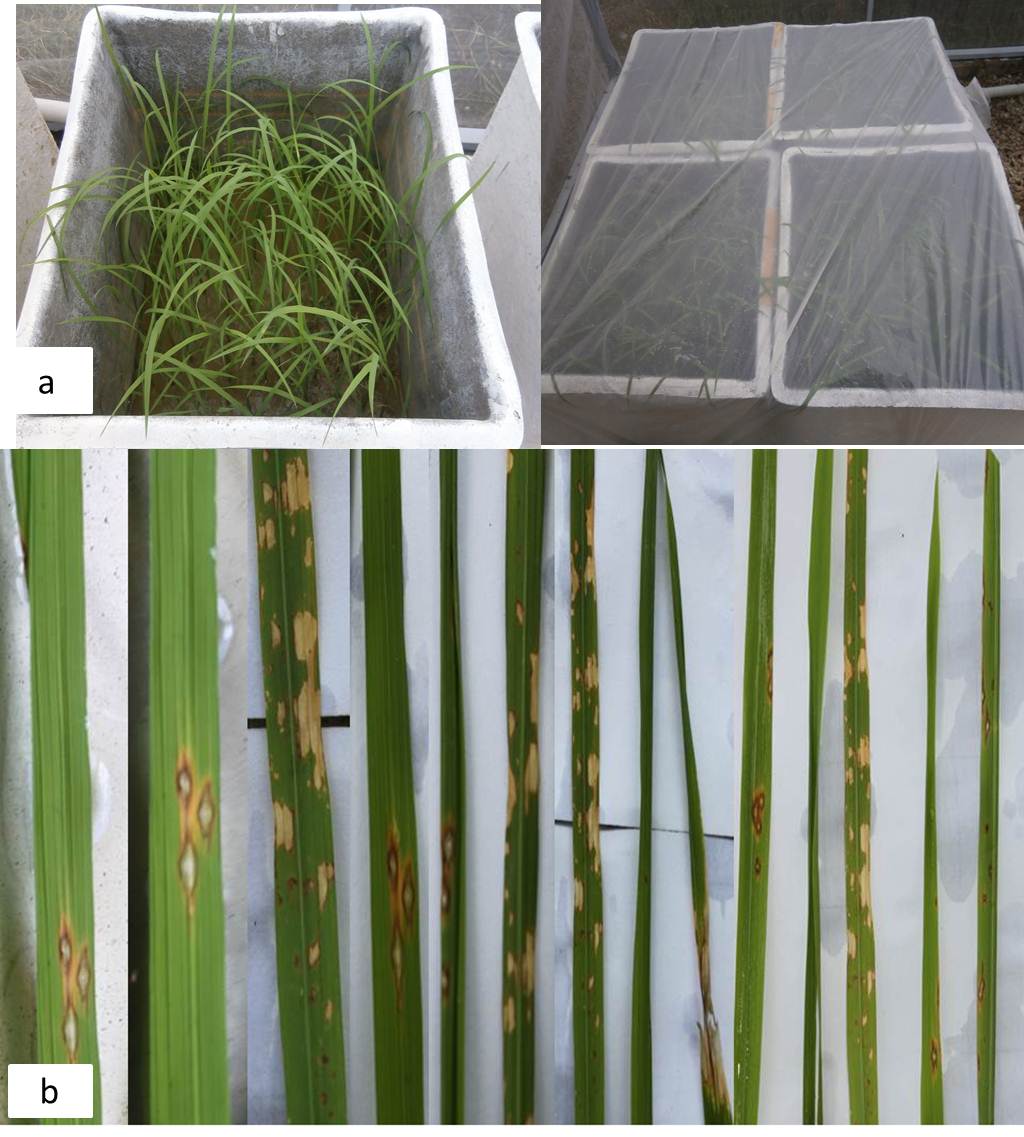

Supplement: Supplementary file 3 [file Image_2.JPEG]
